# GAD65Abs Are Not Associated With Beta-Cell Dysfunction in Patients With T2D in the GRADE Study

**DOI:** 10.1210/jendso/bvad179

**Published:** 2024-02-08

**Authors:** Christiane S Hampe, Ali Shojaie, Barbara Brooks-Worrell, Sepideh Dibay, Kristina Utzschneider, Steven E Kahn, Mary E Larkin, Mary L Johnson, Naji Younes, Neda Rasouli, Cyrus Desouza, Robert M Cohen, Jean Y Park, Hermes J Florez, Willy Marcos Valencia, Jerry P Palmer, Ashok Balasubramanyam

**Affiliations:** Immusoft, Seattle, WA 98103, USA; Department of Biostatistics, Department of Medicine, University of Washington, Seattle, WA 98185, USA; Department of Biostatistics, Department of Medicine, University of Washington, Seattle, WA 98185, USA; Department of Medicine, VA Puget Sound Health Care System, Seattle, WA 98108, USA; Department of Biostatistics, Department of Medicine, University of Washington, Seattle, WA 98185, USA; Department of Biostatistics, Department of Medicine, University of Washington, Seattle, WA 98185, USA; Department of Medicine, VA Puget Sound Health Care System, Seattle, WA 98108, USA; Department of Biostatistics, Department of Medicine, University of Washington, Seattle, WA 98185, USA; Department of Medicine, VA Puget Sound Health Care System, Seattle, WA 98108, USA; Massachusetts General Hospital Diabetes Center, Harvard Medical School, Boston, MA 02114, USA; International Diabetes Center, Minneapolis, MN 55416, USA; The Biostatistics Center, Department of Biostatistics and Bioinformatics, Milken Institute School of Public Health, The George Washington University, Rockville, MD 20852, USA; Department of Medicine, University of Colorado School of Medicine, Aurora, CO 80045, USA; Division of Diabetes, Endocrinology and Metabolism, University of Nebraska and Omaha VA Medical Center, Omaha, NE 68198, USA; Division of Endocrinology, Diabetes and Metabolism, University of Cincinnati and Cincinnati VA Medical Center, Cincinnati, OH 45221, USA; Medstar Health, Hyattsville, MD 20782, USA; Department of Medicine, University of Miami, Miami, FL 33135, USA; Division of Endocrinology, Diabetes and Metabolic Diseases, Medical University of South Carolina, Charleston, SC 29425, USA; Division of Endocrinology, Diabetes and Metabolic Diseases, Medical University of South Carolina, Charleston, SC 29425, USA; Geriatric Research, Education and Clinical Center, Bruce W. Carter Veterans Affairs Medical Center, Miami, FL 33125, USA; Robert Stempel Department of Public Health, College of Health and Urban Affairs, Florida International University, Miami, FL 33181, USA; Department of Biostatistics, Department of Medicine, University of Washington, Seattle, WA 98185, USA; Department of Medicine, VA Puget Sound Health Care System, Seattle, WA 98108, USA; Department of Medicine: Endocrinology, Diabetes and Metabolism, Baylor College of Medicine, Houston, TX 77030, USA

**Keywords:** islet autoantibodies, epitope-specific autoantibodies, anti-idiotypic antibodies, T cell–mediated autoimmunity, humoral autoimmunity, latent autoimmune diabetes of adults

## Abstract

**Context:**

Autoantibodies directed against the 65-kilodalton isoform of glutamic acid decarboxylase (GAD65Abs) are markers of autoimmune type 1 diabetes (T1D) but are also present in patients with Latent Autoimmune Diabetes of Adults and autoimmune neuromuscular diseases, and also in healthy individuals. Phenotypic differences between these conditions are reflected in epitope-specific GAD65Abs and anti-idiotypic antibodies (anti-Id) against GAD65Abs. We previously reported that 7.8% of T2D patients in the GRADE study have GAD65Abs but found that GAD65Ab positivity was not correlated with beta-cell function, glycated hemoglobin (HbA1c), or fasting glucose levels.

**Context:**

In this study, we aimed to better characterize islet autoantibodies in this T2D cohort. This is an ancillary study to NCT01794143.

**Methods:**

We stringently defined GAD65Ab positivity with a competition assay, analyzed GAD65Ab-specific epitopes, and measured GAD65Ab-specific anti-Id in serum.

**Results:**

Competition assays confirmed that 5.9% of the patients were GAD65Ab positive, but beta-cell function was not associated with GAD65Ab positivity, GAD65Ab epitope specificity or GAD65Ab-specific anti-Id. GAD65-related autoantibody responses in GRADE T2D patients resemble profiles in healthy individuals (low GAD65Ab titers, presence of a single autoantibody, lack of a distinct epitope pattern, and presence of anti-Id to diabetes-associated GAD65Ab). In this T2D cohort, GAD65Ab positivity is likely unrelated to the pathogenesis of beta-cell dysfunction.

**Conclusion:**

Evidence for islet autoimmunity in the pathophysiology of T2D beta-cell dysfunction is growing, but T1D-associated autoantibodies may not accurately reflect the nature of their autoimmune process.

Beta-cell dysfunction is critical to the pathophysiology of all forms of diabetes [1-6]. Whereas islet autoimmunity is considered the predominant cause of beta-cell dysfunction in patients with type 1 diabetes (T1D) [2, 3], it traditionally has not been considered a cause of beta-cell dysfunction in type 2 diabetes (T2D); rather, inflammatory and metabolic factors, as well as chronic stress, leading to de-differentiation or transdifferentiation have been invoked to explain defects in islet cell mass or function in T2D [1, 4-6] Growing evidence points to phenotypic and genotypic heterogeneity, due in part to varying pathophysiological mechanisms of beta-cell dysfunction, among patients diagnosed with T2D [7, 8]. Emerging data suggest that islet autoimmunity, associated with deficient insulin secretion, may also develop in many patients with T2D, blurring the distinction between T1D and T2D [8]. One example of a pathophysiologic overlap between T1D and T2D is Latent Autoimmune Diabetes of Adults (LADA), wherein patients with an initial diagnosis of T2D manifest islet autoantibodies typical of T1D, associated with an earlier requirement for insulin therapy [9]. We recently showed, in a cohort of T2D patients in the Glycemia Reduction Approaches in Diabetes—a Comparative Effectiveness (GRADE) Study, that 13.5% of patients had one or more T1D-associated islet cell autoantibodies (7.8% with autoantibodies directed against the 65 kDa glutamate decarboxylase [GAD65Ab]) while 41.3% had islet autoimmunity defined by T-cell autoreactivity to islet antigens [10]. Only 5.4% had both these humoral and cellular markers of islet autoimmunity, suggesting the existence of a distinct group of T2D patients with T cell–mediated cellular islet autoimmunity and another with T1D-associated humoral islet autoimmunity. Importantly, T-cell reactivity correlated inversely with beta-cell function, whereas the presence of T1D-associated islet autoantibodies did not correlate with beta-cell function in these patients [10]. These findings raise questions regarding the relevance of T1D-associated autoantibodies as markers for islet autoimmunity in T2D patients. Indeed, earlier reports suggest that islet autoantibody specificities differ between T1D and T2D patients [11, 12]. Furthermore, the presence of a single T1D-associated autoantibody does not per se denote a significant risk of developing T1D in healthy adults [13] and children [14]. It is therefore possible that patients diagnosed with T2D and presenting T1D-associated islet autoantibodies (especially with just a single T1D autoantibody) might either have LADA and suffer progressive autoimmune-mediated destruction of beta cells or they may have T2D without autoimmune-mediated loss of beta-cell function.

Currently, a T1D-associated autoimmune etiology is inferred by autoantibodies directed against GAD65, islet antigen 2 (IA2), insulin, or zinc transporter 8 (ZnT8) [15] and the absence of anti-idiotypic antibodies (anti-Id) directed against GAD65Ab [16]. GAD65Ab anti-Id mask the presence of GAD65Ab, and there is a specific lack of anti-Id that mask the disease-associated GAD65Ab epitopes in patients with autoimmune T1D [16]. GAD65Ab epitope specificities differ significantly between T1D patients, LADA patients, and healthy individuals [17]. Hence, in GAD65Ab-positive persons, GAD65Ab epitope specificity can differentiate between these phenotypes and provide a pathophysiologic correlation of the circulating autoantibodies with beta-cell dysfunction in each case.

In the present study, we used these distinct characteristics to investigate the humoral autoreactivity to GAD65 in our cohort of patients with T2D in the GRADE study. We evaluated beta-cell function with in-depth analyses of T1D-associated humoral autoimmunity in their serum samples, beginning with a stringent definition of GAD65Ab positivity using a competition assay and proceeding to investigations of GAD65Ab epitope specificities and GAD65Ab-specific anti-Id.

## Materials and Methods

### Study Cohort

GRADE was a 36-center randomized clinical trial evaluating the effectiveness of the addition of 4 classes of glucose-lowering medications to metformin in patients with T2D [18-20]. Prospective GRADE participants went through a run-in period when the metformin dose was increased to 2 g/d with the requirement of a maximal tolerated dose ≥ 1 g/d; 5047 adults with glycated hemoglobin (HbA1c) levels of 6.8% to 8.5% at the end of run-in were randomized and underwent baseline testing including an oral glucose tolerance test (OGTT) [18]. The Beta Cell Ancillary Study was nested within GRADE to measure humoral and cellular islet autoimmunity in baseline blood samples and determine their relationship to beta-cell function measurements derived from the OGTT. All GRADE clinical centers were invited to participate. Nineteen centers obtained local Institutional Review Board approval for the ancillary study and contributed participants.

#### Subjects

Any participant randomized into the parent GRADE study (ClinicalTrials.gov ID: NCT01794143) was eligible for this study [18, 19]. Briefly, patients diagnosed with T2D at age ≥ 30 years (≥ 20 years for American Indians), diabetes duration < 10 years, on metformin ≥ 1000 mg/d at the participating centers, were invited to participate. Key exclusion criteria were: clinical suspicion of T1D, treatment with any glucose-lowering medication other than metformin in the previous 6 months, major cardiovascular events in the previous year, planning pregnancy during the course of the study, heart failure, pancreatitis, cancer, serum creatinine > 1.4 mg/dL in women or > 1.5 mg/dL in men, liver disease or alanine aminotransferase > 3× upper limit of normal, alcoholism, glucocorticoid or antipsychotic use, and conditions rendering HbA1c results unreliable. There were no additional eligibility criteria for the Beta Cell Ancillary Study. The 19 GRADE centers participating in this ancillary study recruited 419 T2D participants (representing 8.3% of the overall GRADE cohort).

#### Sample collection

Blood samples were collected during the baseline OGTT, when metformin was the sole glucose-lowering medication for all participants. Fasting blood was collected into heparin-coated tubes and shipped overnight from the clinical site to Seattle for processing. Plasma was separated from 5 mL blood and frozen at −80 °C for the autoantibody assays. The remaining blood sample was used for the islet-specific T-cell assay. Samples collected at each OGTT time point were used to measure glucose and C-peptide by the GRADE central laboratory (University of Minnesota, Minneapolis, MN). Of the 419 GRADE participants who gave informed consent and provided baseline samples, autoantibodies could not be measured in 27 samples because of insufficient volume or severe hemolysis. Seventy samples could not be included in the T-cell assay because of delayed delivery, insufficient volume, nonviability of peripheral blood mononuclear cells (PBMCs), or severe hemolysis.

### GAD65Ab Radioligand Binding Assay

We measured autoantibodies to 65 kDa glutamate decarboxylase antigen (GAD65Ab) in 392 plasma samples using a radioligand binding assay as previously described [10]. To confirm GAD65Ab positivity, we performed a competition assay employing recombinant human GAD65 (rhGAD65) (Diamyd Medical, Stockholm, Sweden) as previously described [21]. The samples were incubated with radiolabeled GAD65 in the absence or presence of rhGAD65 (200 ng/mL). Samples in which binding to radiolabeled GAD65 was reduced by 40% in the presence of rhGAD65 were considered to be positive for GAD65Ab [21].

### GAD65Ab Epitope-Mapping Assay

Monoclonal GAD65Abs used in this study included human antibodies DPA, DPD, b96.11 and b78, and mouse monoclonal antibody N-GAD65-Ab, as previously described [17]. Human monoclonal antibody HAA1 (ATCC Manassas VA, USA, ATCC number: HB-8534) is directed against blood group A antigen and served as a control. Fab fragments of monoclonal GAD65Ab were cloned and expressed as described [17]. The capacity of GAD65-specific rFab to inhibit GAD65 binding by human serum GAD65Abs was tested in a competitive radioligand binding assay [17]. Binding of GAD65Ab to GAD65 in the presence of rFab was expressed as counts per minute of sulfur-35-labeled GAD65 ([S35]GAD65) bound in the presence of rFab/counts per minute of [S35]GAD65 bound in the absence of rFab × 100. The cutoff for specific competition was > 15%, as determined by control rFab HAA1. In a few cases, the rFab-competed sample resulted in higher counts per minute than the non-competed sample; this was attributable to intra-assay variations.

### Anti-Id to GAD65Ab

The complexes of GAD65Ab and anti-Id in serum samples were dissociated as described previously [16]. Anti-Id was calculated as the observed increase in GAD65Ab levels after absorption compared with the GAD65Ab level before absorption (index after absorption—index before absorption) as previously reported [16]. Antibody levels were expressed as a relative index to correct for inter-assay variation using an in-house serum sample obtained from a healthy donor.

### Beta-Cell Function Analysis

Beta-cell function was assessed utilizing plasma C-peptide and glucose measurements obtained at all sampling timepoints in the baseline GRADE OGTT as previously reported [10]. Two measures of beta-cell function were calculated (after adjusting for insulin sensitivity): ratio of the incremental area under the curve (iAUC) of C-peptide to the iAUC of glucose from 0-120 minutes (iAUC-CG, nmol/mg) adjusted for insulin sensitivity, with iAUC calculated using the trapezoidal rule [22] and ratio of the increment of C-peptide to that of glucose over the first 30 minutes (C-peptide index: [ΔC-peptide (0-30 min)/Δglucose (0-30 min)], nmol/g) [23]. Insulin sensitivity (to adjust the beta-cell response) was estimated using 1/fasting C-peptide or C-peptide–based Homeostasis Model Assessment of steady-state insulin sensitivity (HOMA2-S, obtained using the HOMA2 Calculator version 2.2.3 [Diabetes Trials Unit, University of Oxford, Oxford, UK]) [24, 25].

### Statistical Analysis

Correlations between antibody levels were investigated with Spearman's rank correlation test. In addition to investigating the marginal associations between autoantibodies and measures of beta-cell function, linear models with log-transformed measures of beta-cell function as the outcome and autoantibody status as exposure of interest were also used to assess the association after adjusting for the relevant prespecified covariates. Either HOMA2-S or 1/fasting C-peptide was used to adjust the beta-cell response for insulin sensitivity and the models were also adjusted for status of T-cell reactivity to islet antigens. Covariates including age, sex, body mass index (BMI), duration of diabetes, and medications other than metformin, were prespecified and used to adjust regression models for hypotheses involving autoantibodies or T-cell reactivity and beta-cell function. Details of the models, covariates, and methods of handling of missing values have been previously described [10]. The original sample size for this study was determined to achieve 90% power for detecting differences in beta-cell function among T+ and T− patients in the longitudinal GRADE study without adjustment for additional covariates [10].

## Results

### Participant Demographics

As previously reported [10], the 419 participants enrolled in the GRADE Beta Cell Ancillary Study were 33.9% female, aged 57.4 ± 10.1 years, with BMI 33.6 ± 6.2 kg/m^2^, diabetes duration 4.0 ± 3.0 years, and HbA1c 7.5% ± 0.5% (mean ± SD). Comprehensive demographic and biochemical data on these participants were previously reported [10] and are shown in Supplementary Table S1 [26]. There were no significant differences in any of the reported parameters between the complete Beta Cell Ancillary Study cohort and the 392 participants whose baseline serum samples were adequate for the GAD65Ab analyses reported below.

### GAD65Ab Positivity by Competition Binding

**Figure 1.**
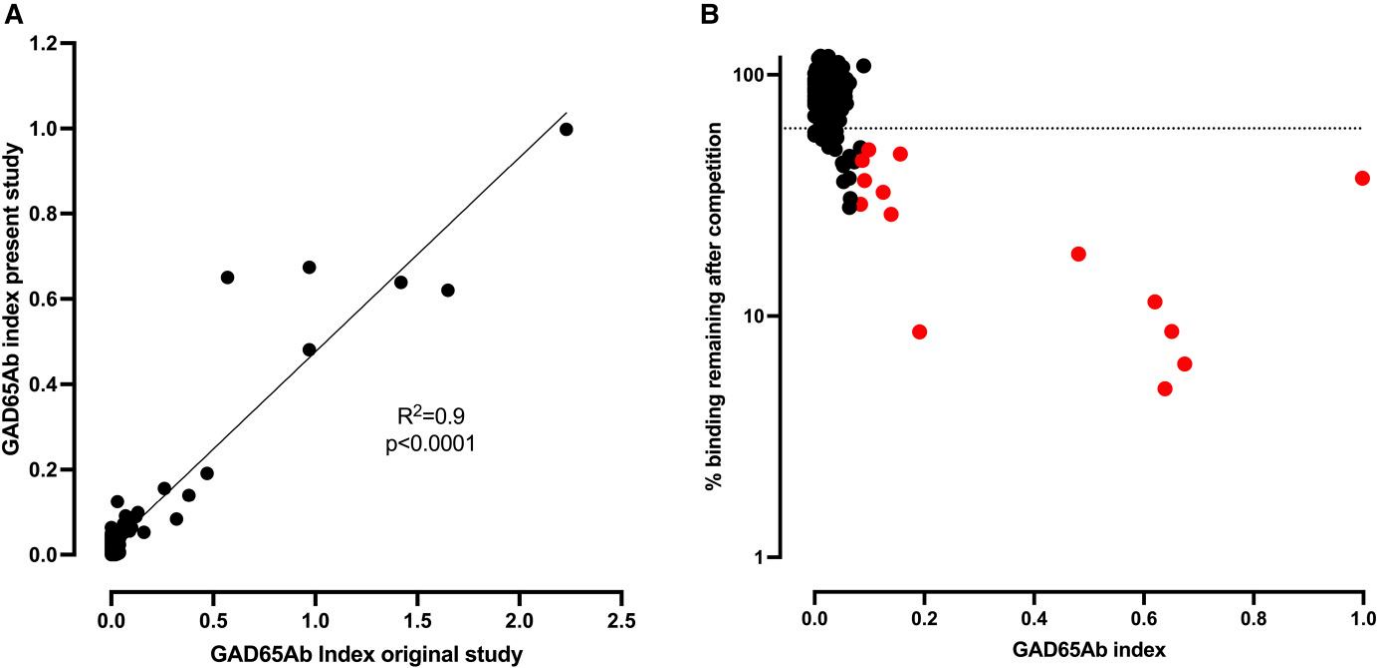
GAD65Ab positivity correlated with competition binding to GAD65. All plasma samples from the GRADE Beta Cell Ancillary Study were re-analyzed for binding to GAD65. a, GAD65Ab indices obtained in the present analysis correlated with those reported previously [10]. The line for simple linear regression is shown together with R-value and *P*-value. b, GAD65Ab positivity was confirmed in a displacement assay using recombinant human GAD65 as a competitor. The cutoff for positivity (40% competition) is indicated by the dashed line. Samples that were subsequently analyzed for GAD65Ab epitopes are indicated by red dots.

All samples (n = 392) were previously analyzed for GAD65Ab titers and the results were reported in our prior publication [10]. GAD65Ab positivity in that report was based on a cutoff at the 98th percentile of titers in an unaffected control population. For the purpose of the present study, all samples were re-analyzed for the presence of GAD65Ab, and GAD65Ab positivity was confirmed in a competition binding assay using nonlabeled recombinant human GAD65 antigen (Fig. 1). GAD65Ab indices of the repeat assay showed significant correlation with those reported previously [10] (*P* < .0001) (Fig. 1A). The median GAD65Ab index was 0.022 and ranged from 0 to 1. The competition binding assay confirmed GAD65Ab-positive status in 23 of the 32 samples that were previously reported as GAD65Ab positive. Nine of the 32 samples originally reported to be GAD65Ab positive (based on the 98th percentile cutoff) were found to have been false positive (competition ≤60%). These 9 samples had been borderline positive in the original report [10]. Additionally, we found 6 samples that we previously reported as GAD65Ab negative (based on the 98th percentile cutoff) to have been false negative (competition ≥ 40%). A repeat analysis of possible correlations between GAD65Ab status and the 2 calculated measures of beta-cell function was conducted, but no significant correlation was observed (*P* = .47 for iAUC-CG and *P* = .44 for C-peptide Index).

### Epitope Analysis

**Figure 2.**
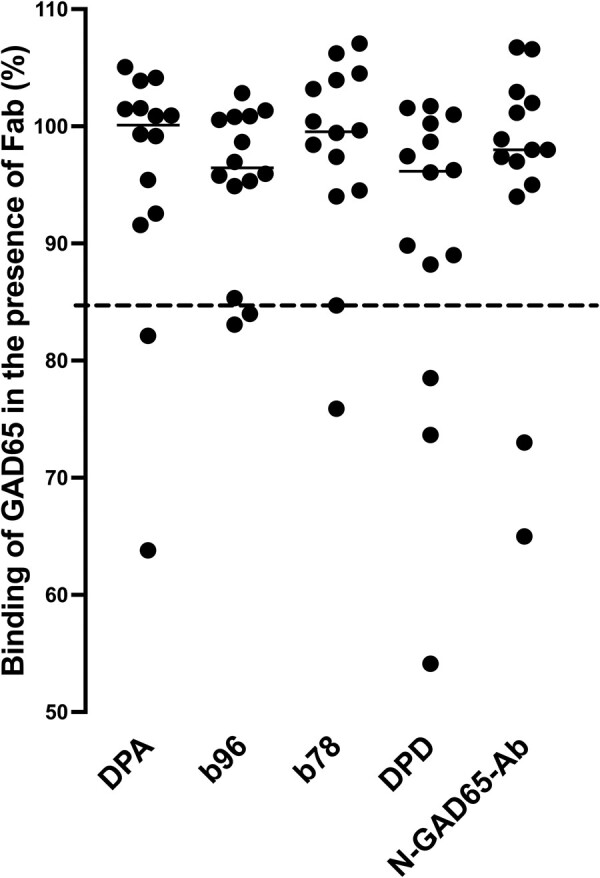
GAD65Ab epitope pattern in 14 GAD65Ab-positive T2D patients does not resemble that found in T1D patients or LADA patients. Binding of serum samples to GAD65 was evaluated in the presence of rFab DPA, b96.11, b78, DPD, and N-GAD65-Ab, and is reported as the percentage of uncompeted binding (set at 100%). The percentage bound remaining after competition with each rFab is presented for each sample. Short solid horizontal lines indicate median binding to each epitope. The cutoff value for successful competition is indicated by the dashed horizontal line. (Note: some dots directly overlie others in the DPD and N-GAD65-Ab columns.) The data indicate that the GAD65Ab epitope patterns in these GAD65Ab-positive T2D patients does not resemble those typically found in T1D or LADA patients.

The underlying autoimmune response in different GAD65Ab disease phenotypes is reflected in specific GAD65Ab epitope recognition [17, 27]. GAD65Abs in patients diagnosed with T1D predominantly recognize an epitope shared with monoclonal GAD65Ab b96.11 [17], whereas GAD65Abs in patients with neuromuscular autoimmune diseases recognize epitopes shared with b78 and N-GAD65-Ab [27], those in patients diagnosed with LADA recognize the DPD-defined epitope [17] and those in healthy individuals recognize the DPA-defined epitope [17]. To further investigate the autoimmune response associated with GAD65Ab in this study, an epitope analysis was performed in those samples that had adequate antibody titers (n = 14 of the 23 competition-confirmed GAD65Ab-positive serum samples, indicated as red dots in Fig. 1B). Antibody titers of the remaining 9 samples were too low (GAD65Ab index ≤ 0.05) to permit reliable epitope analysis. We showed previously that the signal-to-noise ratio is attenuated in serum samples with such low GAD65Ab titers, diminishing the accuracy of our epitope-mapping assay based on competition with recombinant Fab [28, 29]. Epitope analysis data are displayed in Fig. 2.

Two samples each shared a GAD65Ab epitope with monoclonal GAD65Ab b96.11, b78, DPA, and N-GAD65-Ab, and 3 samples each shared a GAD65Ab epitope with monoclonal GAD65Ab DPD. Beta-cell function (iAUC-CG or CpepIndex) in patients with GAD65Ab pattern associated with T1D (recognition of an epitope shared with monoclonal GAD65Ab b96.11) or LADA (recognition of an epitope shared with monoclonal GAD65Ab DPD) did not differ significantly from that in patients, whose GAD65Ab did not recognize these epitopes (data not shown).

### Anti-Id Levels Specific to GAD65Ab b96.11 and DPD

**Figure 3.**
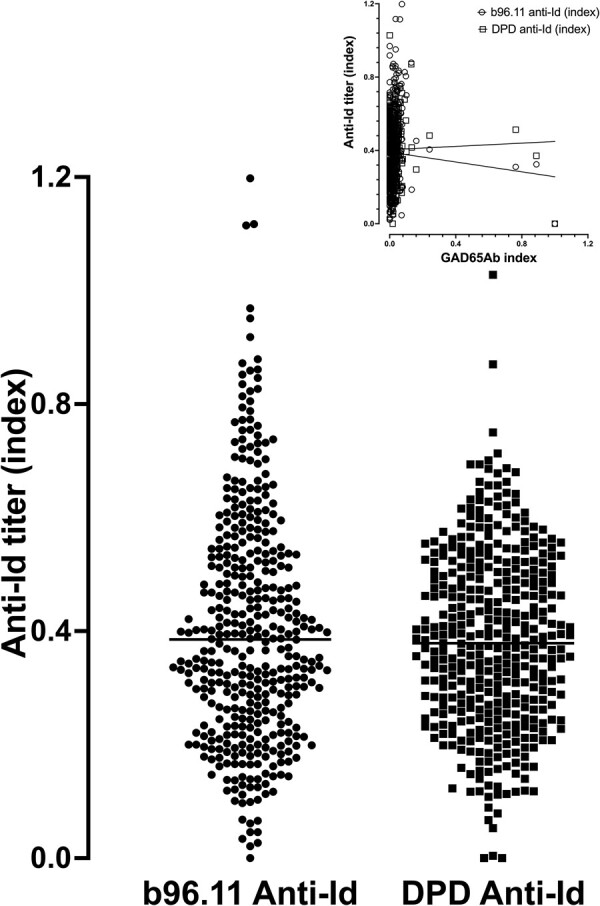
Anti-Id levels specific to monoclonal GAD65Ab b96.11 and DPD. B96.11 and DPD-specific anti-Id titers were analyzed in all samples. Anti-Id titers expressed as an index for each individual subject are shown. Median anti-Id titer index is indicated by horizontal lines. Insert shows correlations between b96.11-specific anti-Id and DPD-specific anti-Id with the GAD65Ab index. There is no relationship between epitope-specific anti-Id and GAD65Ab levels.

A notable aspect of the humoral islet autoimmunity of patients with T1D is the relative lack of anti-Id directed against GAD65Ab, specifically toward GAD65Abs that recognize the b96.11 and DPD epitopes [16]. Hence anti-Id levels specific to GAD65Ab b96.11 and DPD were analyzed for all serum samples. The results are shown in Fig. 3.

Median levels for b96.11- and DPD-specific anti-Id were 0.38 and 0.37, respectively, with ranges of 0 to 1.2 and 0 to 1, respectively. No difference between b96.11- and DPD-specific median anti-Id indices was observed. Levels of b96.11-specific anti-Id correlated with those of DPD-specific anti-Id (*P* < .0001, *r* = 0.26). Neither b96.11-specific anti-Id nor DPD-specific anti-Id correlated with GAD65Ab levels (Fig. 3, insert).

### Anti-Id Levels and Beta-Cell Function.

The presence of anti-Id to GAD65Ab could indicate protection against humoral islet autoimmunity, hence levels of anti-Id might correlate directly with beta-cell function. We examined 2 epitope-specific anti-Id that have been shown previously to associate with specific phenotypes of autoimmune diabetes: anti-Id to b96.11, which is absent in patients with T1D [16], and anti-Id to DPD, which is significantly higher in patients with Ketosis-Prone Diabetes who have preserved beta-cell function compared to those without preserved beta-cell function [30].

**Figure 4.**
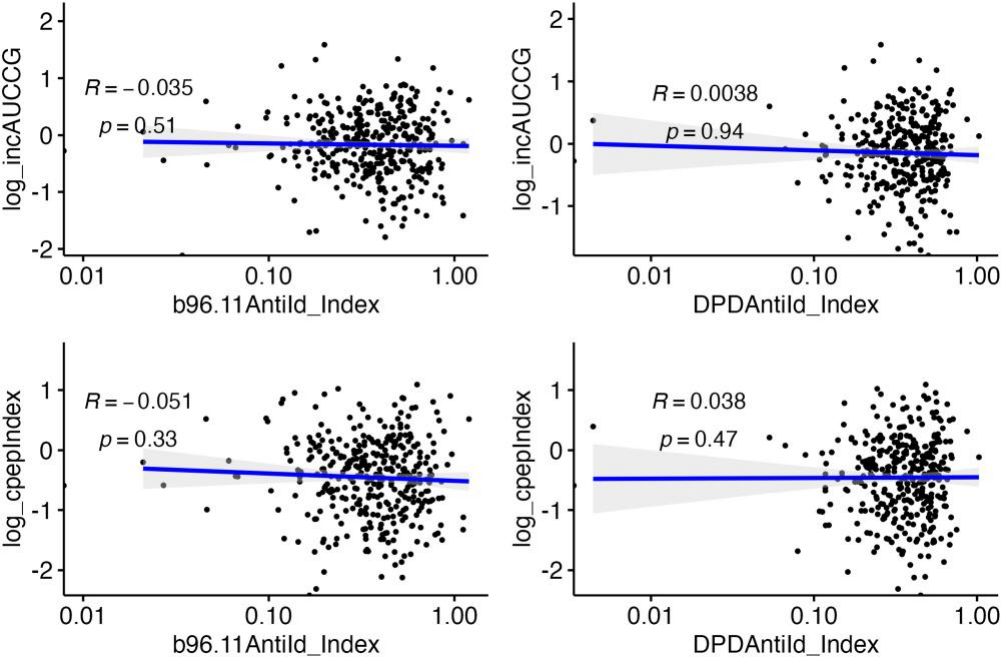
Anti-Id levels do not correlate with beta-cell function. The plots display the relationship of anti-Id specific for b96.11 and DPD with 2 measurements of beta-cell function calculated from OGTT data (iAUC-CG: upper panels, and C-peptide index: lower panels). There is no correlation between anti-Id levels and beta-cell function. (Note: The analyses were repeated after removing the lowest 3 values for the anti-Id index from each of the plots, and all correlations remained nonsignificant; see Supplementary Fig. S1 [26].) There is no correlation between anti-Id levels and beta-cell function.

Hence, we tested correlations of levels of anti-Id directed toward these 2 GAD65 epitope-specific antibodies against the 2 calculated values for beta-cell function (Fig. 4). The 2 epitope-specific GAD65 anti-Id levels showed no significant relationship with either measure of beta-cell function. Similar null correlations were observed for these 2 epitope-specific GAD65 anti-Id levels with calculated measures of beta-cell function in multivariable models that adjusted for relevant covariates as described in “Statistical Analysis” (data not shown).

### Anti-Id Levels and T-Cell Reactivity

**Figure 5.**
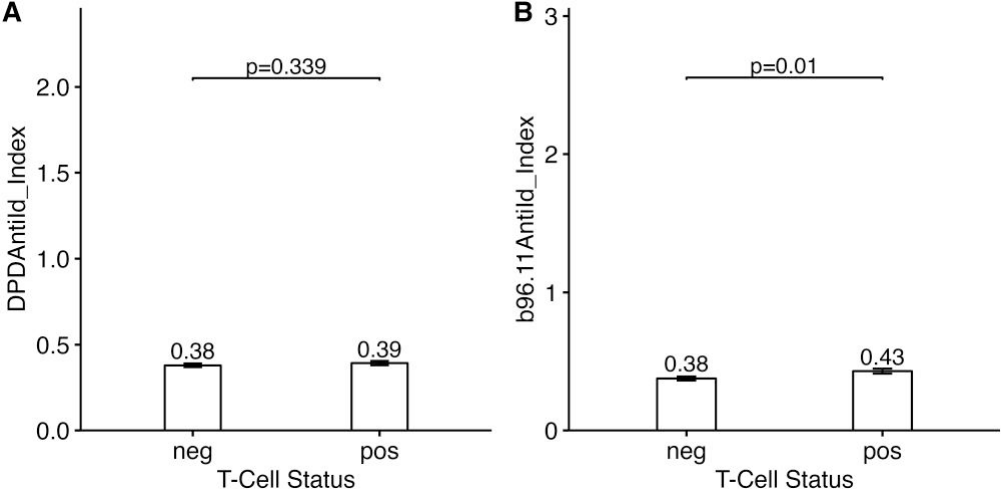
Anti-Id levels are not significantly lower in T cell–positive T2D patients. Anti-Id levels specific for GAD65Ab DPD (A) or b96.11 (B) were compared (using Wilcoxon rank sum test) in sera from blood samples that tested negative or positive for T-cell reactivity against islet antigens. Bars show mean values +/− SE. Anti-Id levels are not lower in the T cell–positive compared to the T cell–negative T2D patients.

Since anti-Id may protect against the development of GAD65Ab-associated humoral islet autoimmunity, we asked whether they might also demonstrate a negative relationship to cellular (T cell–mediated) reactivity to islet antigens. Hence, we interrogated the anti-Id titers for associations with T-cell reactivity as measured in the same participant blood samples and previously reported by us [10] (Fig. 5). No difference in DPD-specific anti-Id titers was observed between T+ compared to T− groups. Titers of anti-Id specific to b96.11 were actually higher in sera from T+ participants compared to sera from T− participants (0.43 vs 0.38, *P* = .01). We also investigated whether anti-Id levels correlated with beta-cell function in T+ or T− patients separately. We observed no significant correlation between either b96.11- or DPD-specific anti-Id levels and either measure of beta-cell function (iAUC-CG or CpepIndex) in T+ or T− patients (data not shown).

## Discussion

Our comprehensive analysis revealed that GAD65Ab positivity is unlikely to be related to autoimmune-mediated beta-cell dysfunction among participants in this substudy of the GRADE T2D cohort. These data provide a foundation for our earlier finding that humoral T1D-associated markers of islet autoimmunity are not associated with beta-cell function, HbA1c, or fasting glucose levels in this cohort [10].

GAD65Ab are present in patients with classic autoimmune T1D, as well as in patients with LADA [31], patients with autoimmune neuromuscular diseases [32] and healthy individuals [33]. The respective underlying autoimmune responses are reflected in GAD65Ab titers, distinct GAD65Ab epitopes and anti-Id directed against epitope-specific GAD65Abs [16, 17]. Our analysis of GAD65Ab in patients with T2D in the GRADE study revealed GAD65Ab patterns that differed significantly from those observed in patients with T1D or LADA, showing low GAD65Ab titers, lack of GAD65Ab epitope specificities characteristic for patients with T1D or LADA, and a wide range of GAD65Ab epitope-specific anti-Id levels, rather than a lack of GAD65Ab epitope-specific anti-Ids as found in patients diagnosed with T1D or LADA [16]. Moreover, anti-Id titers were not associated with beta-cell function, indicating that the presence or titers of anti-Ids cannot define and are not related to specific diabetes phenotypes in this cohort. However, the relatively small sample size and greater variability of OGTT-based calculations of beta-cell function (compared with methods based on intravenous glucose testing) could have made it harder to show such a relationship to anti-Id levels or GAD65Ab positivity based on the competition assay.

In conclusion, the GAD65-related autoantibody response in this cohort resembles GAD65Ab profiles found in healthy individuals, with low GAD65Ab titers, lack of a distinct GAD65Ab epitope pattern, and presence of anti-Id to diabetes-related GAD65Ab. However, the 5.9% frequency of GAD65Ab positivity within this T2D population (23 confirmed GAD65Ab-positive persons among 392 participants) is higher than the reported frequency of about 2% in healthy persons [13]. GAD65Ab positivity in patients diagnosed with T2D may predict insulin dependence within 3 to 5 years [31, 34, 35], although this was not observed in a large longitudinal study [36]. Taken together with the observation that lower beta-cell function or more rapid decline in beta-cell function in LADA patients appears to be observed mainly among patients with high autoantibody titers or multiple autoantibodies [37-40], it is possible that some of our GAD65Ab-positive patients may develop insulin dependence in the future. Previous reports showed that genetic factors driving islet autoimmunity (calculated by T1D genetic risk score) are additive with GAD65Ab positivity to promote a more rapid rate of beta-cell function loss [41, 42]. Thus, the absence of diminished beta-cell function among GAD65Ab-positive patients in our cohort could be caused by a lack of genetic propensity to autoimmune beta-cell destruction. and insufficient follow-up. Moreover, since GRADE entry criteria included T2D patients with a duration of diabetes < 10 years on metformin alone, the parent study may have excluded patients with pathogenetically relevant GAD65Ab positivity, whose beta-cell function and glycemic control declined more rapidly. Conversely, very recently diagnosed participants might not have had diminished beta-cell function when measured at the time of study enrollment, despite being GAD65Ab positive. Follow-up with the arc of beta-cell function over the course of the GRADE study will be important to strengthen this conclusion. Interestingly, 65% of participants whose GAD65Ab positivity was confirmed by competition assay had poorer primary glycemic outcomes compared to the average of their randomized treatment groups in the parent GRADE study, that is, they either reached the prespecified primary outcome of HbA1c ≥ 7% in the time-to-failure analysis earlier than average or their HbA1c levels always remained higher than the initial goal of < 7% (data not shown).

Our results showing lack of association between GAD65Ab positivity and worse beta-cell function in the present cohort differ from previous reports of this association [43-47]. Apart from the fact that these earlier studies did not define GAD65Ab positivity as rigorously as in the present study, reasons for the discrepancy could include: (i) our use of a comprehensive assessment of beta-cell function utilizing the dynamic response of C-peptide to glucose over a 2-hour OGTT, rather than fasting values for glucose and insulin or C-peptide in the previous studies; (ii) the racial/ethnic diversity of our T2D cohort, since the association of islet autoantibody positivity with beta-cell function has been shown to be absent in a study of African American youth with T2D [48]; and (iii) the fact that beta-cell dysfunction has been demonstrated mainly in patients with high islet autoantibody titers [13, 37, 39, 40, 49], which was not the case in our cohort. The relatively small sample size of those we found to be GAD65Ab positive could also have affected our results.

We acknowledge that methods published since the completion of our study allow the detection of disease-relevant high-affinity signals exclusively [50, 51], while the traditional radioligand binding assay employed by us detects both low-affinity and high-affinity GAD65Ab. Such precise assays should be considered for future investigations of the relation of GAD65Ab positivity to beta-cell function in T2D patients.

These data in a carefully phenotyped cohort of T2D patients in the GRADE study demonstrate the inadequacy of a T1D-associated autoantibody marker like GAD65Ab to define pathogenetically relevant islet autoimmunity in T2D. Our T-cell assay tests cellular reactivity to a wide range of islet cell antigens [52], and hence differs significantly from the autoantibody assays that detect humoral responses to restricted autoantigens. Taken together with previous reports of potentially novel islet autoantibodies in patients with T2D [11, 12], the autoimmune responses in T2D patients may differ in antigen specificity from those observed in T1D patients. There is a pressing need to identify distinct antigens recognized by autoreactive T cells among T2D patients, since “T cell–positive” patients in this cohort do manifest islet autoimmunity associated with diminished beta-cell function [10]. Once these are identified, it will be important to develop assays to detect antigen/epitope-specific autoantibodies for better screening of islet autoimmunity to differentiate patients with varying phenotypes and natural histories of T2D.

## Data Availability

Some or all datasets generated during and/or analyzed during the current study are not publicly available but are available from the corresponding author on reasonable request.

## Abbreviations

anti-Id: anti-idiotypic antibodies
GAD65: glutamic acid decarboxylase 65-kilodalton isoform
GAD65Abs: GAD65 autoantibodies
GRADE: Glycemia Reduction Approaches in Diabetes—a Comparative Effectiveness
HbA1c: glycated hemoglobin
HOMA2-S: Homeostasis Model Assessment of steady-state insulin sensitivity
LADA: latent autoimmune diabetes of adults
OGTT: oral glucose tolerance test
T1D: type 1 diabetes
T2D: type 2 diabetes
