# Correction to: “GAD65Abs Are Not Associated With Beta-Cell Dysfunction in Patients With T2D in the GRADE Study”

**DOI:** 10.1210/jendso/bvae058

**Published:** 2024-04-01

In the above-named article by Hampe CS, Shojaie A, Brooks-Worrell B, Dibay S, Utzschneider K, Kahn SE, Larkin ME, Johnson ML, Younes N, Rasouli N, Desouza C, Cohen RM, Park JY, Florez HJ, Valencia WM, Palmer JP, and Balasubramanyam A (*J Endo Society*. 2024; 8(3); doi: 10.1210/jendso/bvad179), there was an error in the authorship.

In the originally published article an author “Robert Stempel” was incorrectly included in the author list with a separate affiliation. The full affiliation of “Robert Stempel Department of Public Health” should have been assigned to the author Willy Marcos Valencia. The Publisher acknowledges the error.

The article has been corrected online.

